# A multi-method exploration into the social networks of young teenagers and their physical activity behavior

**DOI:** 10.1186/s12889-020-10081-0

**Published:** 2021-01-07

**Authors:** Shannon C. Montgomery, Michael Donnelly, Jennifer Badham, Frank Kee, Laura Dunne, Ruth F. Hunter

**Affiliations:** 1 UKCRC Centre of Excellence for Public Health (Northern Ireland)/Centre for Public Health, School of Medicine, Dentistry and Biomedical Sciences, Queen’s University Belfast, Belfast, Northern Ireland, UK; 2 School of Social Sciences, Education and Social Work, Queen’s University Belfast, Belfast, Northern Ireland, UK

**Keywords:** Adolescents, Multi-methods analysis, Physical activity, Social networks

## Abstract

**Background:**

There is a need for novel interventions to target inadequate levels of adolescent physical activity behavior. Previous research indicates that better understanding of the processes by which social networks influence physical activity behavior in adolescents may be useful to enhance intervention design.

**Methods:**

This study used a multi-methods approach to aid our understanding about the role of social networks for adolescent physical activity behavior. The quantitative phase of data collection was analyzed using a three-step linear regression model using cross-sectional data from the WiSe study (*n* = 529 participants, 48.6% female, mean age 14.38 years (SD 0.32)). A demographically reflective sub-sample of schools were invited to take part in the qualitative phase, which involved focus group discussions. Thematic analysis was used to explore findings from the quantitative phase in greater depth, and identify other themes pertaining to the association between social networks and physical activity behavior.

**Results:**

Males’ physical activity behavior was predicted by their *friend group* (0.46, *p* = 0.007) whereas females’ physical activity was predicted by their *best friend* (0.21, *p* = 0.03). The three main findings that were uncovered by the regression analysis were explored during the qualitative phase: 1) friends have similar physical activity behaviors; 2) friendship social networks may influence differently early adolescent male and female physical activity behavior; 3) popularity and sociability were not associated with physical activity behavior. Two additional themes emerged from the analysis of focus group data: 4) social norms and 5) external factors that may impact the relationship between adolescent physical activity behavior and social networks.

**Conclusions:**

The investigation of the interplay between the findings from each phase of the inquiry indicated that social networks influence in different ways and to different degrees the physical activity of adolescent males and females. In turn, these insights point to the need for a systematic tailoring process for the development and implementation of physical activity behavior interventions.

**Supplementary Information:**

The online version contains supplementary material available at 10.1186/s12889-020-10081-0.

## Background

In 2019, the United Kingdom (UK) Government Chief Medical Officers published physical activity guidelines advising that children and young people (aged 5–15 years old) should aim to accumulate a daily average of at least 60 min of moderate to vigorous intensity physical activity (MVPA) [1]. These guidelines concur with the updated Physical Activity Guidelines for Americans, which recommend that children and young people (aged 6–17 years old) should engage in at least 60 min MVPA daily [2]. There has been considerable research spanning many years that demonstrates the many health benefits associated with adhering to the physical activity guidelines [3]. Benefits include cardio-metabolic (e.g. improving physical fitness [4]), physical (e.g. strengthening bones [5]), cognitive (e.g. improved concentration [6]), psychological (e.g. better wellbeing [7]) and social (e.g. develops social skills) [1].

Despite this, it is estimated that less than 20% of adolescents (aged 13–15 years) worldwide are meeting the daily 60 min MVPA [8]. Recent research pooled data from 146 countries worldwide, and estimated that the majority of adolescents are insufficiently physically active [9]. Furthermore, females are significantly less active than males [8]. The British Heart Foundation found that only 8% of females aged 13–15 years old met the physical activity guidelines, compared to 14% of males of the same age in England in 2012 [10]. Recent research showed that between 2001 and 2016, the global prevalence of insufficient physical activity in males decreased by 2.5 percentage points, whereas there was no significant change for females, leading to greater discrepancy between the sexes [9]. There has been a considerable increase in the number of interventions which have focused on increasing adolescent females’ physical activity, but intervention effects have been largely minimal or non-existent [11–15] and the gender disparity remains [16].

The development of novel interventions to address declining physical activity behavior in youths is a high priority in health behavior research [17]. Previous systematic reviews suggest that interventions should utilize peer social networks or friend influence to encourage physical activity behavior in adolescents [18–20]. However, there is a need for better understanding of the association between peer social networks and physical activity behavior in adolescents to inform the development of such interventions. Rarely have adolescent peer social networks been leveraged within physical activity interventions [21]. Social networks refer to a set of individuals with ties, typically friendships, connecting them [22]. Peers are defined as individuals who are similar in age, qualification level, life-stage or maturity. Adolescence is a time of biological and social change for young people, and their health behavior choices are increasingly determined by their social surroundings and peer social networks [23–26] with many individuals experiencing a shift from parental to peer influence [27]. Network ties are often facilitated through social environments, such as the classroom [28–30]. Therefore, much of the research investigating physical activity behavior and peer social networks in the adolescent population has focused on friendship social networks within the boundary of the school or classroom setting. Furthermore, the school or classroom setting is often a favorable setting for adolescent physical activity interventions to be conducted [31], which can be partially attributed to compulsory attendance and convenience of intervention delivery, as Physical Education is already embedded within the curriculum [32, 33]. There is a need for further investigation into peer social networks and adolescent physical activity, to allow for incorporation of social network components within the design of physical activity behavior change interventions [30].

Despite the growing body of evidence that supports the use of peers within adolescent physical activity behavior change interventions to increase physical activity behavior uptake [19], social network approaches are seldom incorporated within adolescent behavior change interventions [21, 34]. However, incorporation of social networks would allow for tailoring of the intervention. For example, adolescent friendships have been shown to be governed by gender homophily (i.e. adolescents tend to befriend individuals of the same sex) [35–38]. Homophily within social networks has been depicted as “birds of a feather flock together” [39] indicating that individuals are more likely to be tied together, for example, through friendships, if they share similar qualities and attributes [37], such as engaging in similar hobbies or spare time interests. A recent systematic review investigated the processes by which peer social networks impact on adolescent health behaviors [30]. This review investigated 55 studies of adolescents (mean age 15.1 years, range 13–18 years, 51.5% female). The limited studies on physical activity behavior demonstrated mixed evidence to support the association between popularity and physical activity behavior, highlighting a need for further investigation into social network processes and physical activity behavior to better understand how social networks impact physical activity behavior. Given the known sex differences in adolescents’ physical activity behavior, there is a need to investigate the role of social networks for both adolescent males’ and females’ physical activity behavior, to identify potential differences in the mechanisms by which social networks could best be used to increase the effectiveness and maintenance of physical activity behavior change interventions.

Further, much of the evidence on social networks and adolescent health behaviors has used social network analysis techniques, which use graph theory to understand the structure and characteristics of social networks [18, 20, 30]. However, this has previously been criticized for its heavy focus on quantitative measures [40], or reliance on evidence gathered cross-sectionally, particularly given that social networks are dynamic and evolve over time [41]. Thus, qualitative exploration of peer social networks could provide rich contextual understanding of how social networks impact physical activity behavior [42]. This would allow for exploration into “deeper relationships” that exist beyond the social network boundary than that which can be explored quantitatively, providing a fuller and more complete appreciation of the role of social networks for physical activity behavior [43]. In summary, research has indicated that peers, and particularly friends, may play an important role in determining adolescent physical activity behavior [18–20]. However, there is a need to further explore the association between such social networks and physical activity behavior, to understand the ways by which social networks impact physical activity behavior and how such interventions might be implemented in practice. Further, research suggests that there may be sex differences in social networks and physical activity behavior [44] that could require different approaches for male and female students.

Therefore, this study used a multi-methods approach within an explanatory sequential design. A multi-methods approach involves initial collection of quantitative data and analysis, followed by collection of qualitative data to elaborate on quantitative findings [45, 46]. Firstly, quantitative data collection and analysis was used to explore the association between adolescents’ physical activity behavior and peer social networks of adolescents aged 13–15 years in a classroom setting. This was followed by a second phase of qualitative data collection, which was used to explore the findings from the first phase. This facilitated a broader and deeper investigation of the potential influence of social networks on physical activity behavior, beyond those which can be quantitatively explored within a bounded network, to provide richer understanding of the findings identified in phase 1 [42, 47].

## Methods

### Study design

This study used a multi-methods cross-sectional design. Phase 1 explored the association between peer social networks and physical activity behavior of adolescent males and females aged 13–15 years old in a classroom setting. Phase 2 involved the conduct of focus groups to provide further explanation of the findings elicited in phase 1 and investigated the role of social networks for physical activity behavior beyond the classroom network.

### Phase 1: quantitative exploration

#### Participants

The study participants were a cross-sectional sample from one wave of the ‘WiSe’ (Well-being in Schools) study [48]. WiSe was a population-wide health and well-being survey involving post-primary schools in Northern Ireland that followed a sample of pupils through their post-primary school journey over three waves between 2013 and 2018. Data was collected biennially and written informed consent was obtained prior to all stages of data collection. Opt-in consent was obtained from participating pupils and opt-out consent was sought from parents/guardians. Ethical approval was sought prior to each wave of data collection due to minor amendments made to the survey (for example, wave 2 included a social networks component that was not included in wave 1). Ethical approval for this study was granted by the School of Education Ethics Committee, Queen’s University Belfast in February 2014. For full details of ethics and consent please see [49].

All post-primary schools in Northern Ireland were invited to take part in the first wave of WiSe data collection (181 schools) and 94 schools agreed to take part. In wave 1, one classroom in Year 8 (age 11–12 years) was randomly selected to participate from each school. Behavioral data was collected on health behaviors including physical activity behavior. Socio-demographic factors included familial financial income, sex and age. Only schools that participated in wave 1 were invited to continue participation in wave 2 (no new schools were recruited). Six schools were lost to follow up at wave 2, resulting in the participation of 88 schools. A social network module was added to the survey in wave 2 (Year 10, age 13–14 in 2015). Therefore, the current study includes analyses of data from a sub-sample of classes from wave 2 only. The schools selected for Phase 1 were a representative sample of the participating schools in WiSe wave 2. Schools were included from all education sectors in Northern Ireland (grammar or secondary school types, Catholic, Protestant or integrated schools), single-sex and mixed schools, geographical regions and levels of deprivation. Further detail regarding the demographic characteristic of the WiSe wave 2 sample (alongside the representativeness of the Phase 1 sub-sample) is included in Appendix file 1, Table 1.

**Table 1:** School demographic characteristics (phases 1 and 2)

Phase 1																								
School		**1**	**2**	**3**	**4**	**5**	**6**	**7**	**8**	**9**	**10**	**11**	**12**	**13**	**14**	**15**	**16**	**17**	**18**	**19**	**20**	**21**	**22**	**23**
Class sex	All-male								X		X	X						X				X		
	All-female	X	X							X														
	Co-educational (% female)			X (47.4)	X (52.4)	X (45.8)	X (45.5)	X (54.2)					X (52.2)	X (51.7)	X (50)	X (50)	X (44.4)		X (66.7)	X (42.3)	X (70)		X (56.5)	X (66.7)
School type	Grammar	X	X	X		X	X	X	X	X	X	X	X			X	X	X			X	X	X	
	Secondary				X									X	X				X	X				X
Deprivation level	More deprived				X										X					X				
	Less deprived	X	X	X		X	X	X	X	X	X	X	X	X		X	X	X	X		X	X	X	X
School Management Type	Voluntary	X	X	X		X			X	X	X	X	X			X	X					X	X	
	Controlled						X	X						X							X			X
	Catholic Maintained				X										X			X	X	X				
Phase 2																								
School		**A**					**B**					**C**				**D**					**E**			
Class sex	All-male	X														X								
	All-female						X					X												
	Co-educational (% female)																				X (50.4)			
School type	Grammar	X					X					X												
	Secondary															X					X			
Deprivation level	More deprived															X								
	Less deprived	X					X					X									X			
School Management Type	Voluntary	X					X					X												
	Controlled															X								
	Catholic Maintained																				X			

Northern Irish schools differ from schools in the United Kingdom or Republic of Ireland. A selection exam during the final year of primary school determines a Grammar or Secondary post-primary school place. Deprivation is based on the % of pupils in the school eligible for free school meals (FSM) (less deprived < 37.5% of pupils are eligible for FSM; more deprived > 37.5% eligible for FSM). Children are eligible for FSM if their parents receive income support, or their net earnings do not exceed £14,000/year. Schools are governed by four main types of management: voluntary (managed by a Board of Governors (BoGs)) controlled (managed and funded by the Education Authority through school BoGs, majority Protestant), Catholic Maintained (managed by BoGs nominated by trustees, mainly Roman Catholic) and Grant Maintained Integrated (managed by a BoGs consisting of trustees or foundation governors, both Catholic and Protestant)

(Dep. of Education 2018 [50])

#### Social network measurement

Friendship social networks were measured within the classroom via free re-call. The social network question was, *“please name up to 10 of your closest friends in your school form class.”* Pupils normally begin the school day in their form classes, where attendance is recorded, therefore, the form class was chosen to be the boundary that pupils would indicate their friendship social networks nominations within. Pupils often remain in the same form class throughout school, however this may vary from school to school. Participants were reminded to only list friends in the school form class, to provide the full name of their nomination (i.e. first name and surname) and that they did not have to provide ten names.

Nominated friends were assigned a unique identification number from the class lists by an independent researcher (author blinded for review). Friend nominations that did not match any names on the class lists or were unidentifiable were excluded from the analyses (see Missing data). Twenty-nine schools (33% of total sample of schools included in wave 2) met the criteria of at least 80% complete network data (i.e. at least 80% of the social network nominations were identifiable). The research team decided this criterion to ensure data quality, based on the criteria for Stochastic Actor Based Models [51]. To enable appropriate network comparisons to be made, classes that were within 1 standard deviation (SD) of the mean class size (19.6, SD 0.5) were selected. Twenty-three schools met the completeness and class size sampling criteria (*n* = 529 pupils), which were inclusive of the demographic characteristics of schools included in the total WiSe sample.

#### Physical activity behavior measurement

The Physical Activity Questionnaire for Children (PAQ-C) [52] was used to measure physical activity behavior in this cohort. The PAQ-C is a validated and reliable self-reported questionnaire which assesses general physical activity levels over the past 7 days, including during-school and after-school periods, and weekends. The PAQ-C comprises nine items, each scored on a 5-point Likert scale, from which a mean physical activity score (PAQ-C summary) is derived. The minimum value of 1 indicates low physical activity behavior, whereas a maximum score of 5 indicates high physical activity behavior.

To date there is no consensus regarding appropriate cut-off points employed to PAQ-C scores to categorize physical activity behavior levels. Due to the lack of consensus and variability in PAQ-C cut-off point recommendations, this study used the scale as a continuous outcome variable (measured on a continuous 1–5 scale, computed to two decimal places), similar to that which has been adopted in other studies [53, 54] and employed linear regression methods. As form classes were selected for the study and the friendship nominations were bound within form classes, the physical activity behavior could be calculated for individuals’ nominated friends, to give the mean physical activity behavior of their nominated friend group. Although the WiSe questionnaire required participants to indicate their best friends with an asterisk, this question was not well answered, with only 32.5% (172/529) indicating a best friend who could be identified from the dataset. Therefore, ‘best friend’ was defined as the participant’s first nominated friend, a strategy which has been adopted successfully in previous research, such as studies from the EAT-2010 (Eating Among Teens) survey [55, 56]. A second separate variable was derived for the physical activity behavior for the nominated best friend.

#### Statistical analyses

This phase aimed to investigate the association between individual physical activity behavior and best friend’s physical activity behavior (physical activity behavior of the individual’s first nominated friend only), or friend group’s physical activity behavior (mean physical activity behavior of the individual’s nominated friends). Additionally, specific network processes of popularity (calculated by in-degree, the number of nominations the individual receives from other individuals in the social network [57]) and sociability (out-degree, the number of friendship nominations an individual makes to other individuals in the social network [58]) were investigated as predictors of physical activity behavior.

UCINET (version 6.0) (Borgatti, S. P., Everett, M. G., & Freeman, L. C. Ucinet for Windows: Software for Social Network Analysis. Harvard, MA.: Analytic Technologies, 2002) specialist social network analysis software, was used to calculate the egocentric (i.e. individual level) measures of out-degree (sociability indicator) and in-degree (popularity indicator). Pearson’s correlation coefficient was used to investigate the strength of the relationship between individual and friend (best friend and friend group) physical activity behavior. All correlation and regression analyses were split by sex, with males and females analyzed separately. Given the differences in adolescent male and female physical activity [8], and evidence of gender homophily in our data, single sex analysis allowed for exploration into the potential social network processes driving physical activity behavior in males and females.

A three-step linear regression model was used to explore the association between individual and friend physical activity behavior. In the first (univariate) model, four independent variables: friend group physical activity behavior, best friend’s physical activity behavior, out-degree and in-degree were investigated as potential predictors of individual (adolescent’s own) physical activity behavior (dependent variable). The second (multivariate) model had all four variables added simultaneously. The third model adjusted for classroom level clustering, and was conducted using STATA version 14.0 (Stata Statistical Software. College Station, TX: StataCorp LP., 2015), as the participants came from 23 separate school classroom networks. With the exception of the third model, all regression analyses were conducted using SPSS version 22.0 (SPSS Statistics for Windows. Armonk, NY: IBM Corp, 2013). The significance level for including a variable in the next model was set as 0.05.

#### Missing data

The sample of 23 schools included 529 participants. Four participants did not provide any physical activity behavior data and were removed from the analyses. A total of 3312 friendship nominations were made, of which 9.8% (324 of 3312) were unidentifiable (i.e. the friendship nominations could not be matched to the class lists) and removed from further analysis. In total, 90.2% of the network data was identifiable and could be matched to unique identification numbers and physical activity behavior data.

### Phase 2: qualitative phase

As the qualitative phase was additional to the WiSe study, specific ethical approval was sought. Ethical approval was granted from the School of Medicine, Dental and Biomedical Sciences Research Ethics Committee at Queen’s University Belfast in November 2017 (reference number: 17.46_v3).

#### Participant recruitment

A maximum variation sampling framework was used to invite a demographically representative sample of schools, based on the Northern Irish school system (i.e. including single-sex and mixed-sex schools, deprivation levels, grammar and secondary education, and rural and urban locations) that participated in the WiSe study to take part in focus group discussions. A study invitation outlining the purpose of the study, the participant requirements and informed consent information was posted to the head-teacher of each school included in the sample in December 2017 by (SM). A participant information sheet was also included, which detailed the purpose of the study, the participant requirements and informed consent information. A follow up phone call was held between the contact teacher and (SM), to further advise on study details, including teacher and pupil requirements for participation. Teachers were provided with an opportunity to ask any questions they had about the study.

If the head-teacher agreed to the participation of their school, a study pack was posted to the school by (SM), which contained pupil information sheets, pupil consent forms, parent/guardian information sheets and parent/guardian opt-out consent forms. The contact teacher (mainly the year group head teacher) was asked to select 8–10 pupils who they perceived to have varying physical activity behavior (i.e. a mixed group of low, moderate and highly physically active pupils), to ensure that recruited participants were representative of the general physical activity behavior of the pupils. Selected participants were provided with the study pack. It was thought that the year group head teacher would be able to identify pupils of varying physical activity behavior (i.e. pupils who were part of sports teams compared to pupils who were less physically active). Those pupils who returned completed consent forms from parents/guardians were eligible to take part. Focus groups were conducted between December 2017 and March 2018, until the research team was satisfied that data saturation was achieved (during focus group five). The schools included two all-male, two all-female and one mixed-sex school. Demographic characteristics of all five schools are detailed in Table 1.

#### Research process

A semi-structured topic guide was developed to further explore findings identified from phase 1, to explore the role of social networks for physical activity behavior. The topic guide was refined iteratively by discussion with the research team (SM, RH, MD) after each focus group to ensure adequate coverage of key issues. The topic guide covered young peoples’ views about physical activity behavior (i.e. including what encourages them to be more active), how friends, family and other social networks might help to encourage physical activity behavior (i.e. how friends or family might help adolescents to be more active) and particularly focused on the perceived importance of friends for encouraging physical activity behavior (i.e. physical activity behavior of classmates, peers or friends, and how these individuals encourage or discourage physical activity behavior).

Before commencing the focus group session, the procedure was explained to all participants and verbal consent (in addition to the written consent) was obtained. Two audio-recorders were switched on prior to commencing the discussion and stopped at the end of the discussion. The discussion lengths had a mean duration of 31 min (range 27–38 min).

#### Data analysis

Transcripts were analyzed using an inductive six-step thematic analysis approach described below [59]:

1. Initially, researchers (SM and RH) familiarized themselves with the data. Transcripts were read comprehensively, supplemented by the audio-recordings to resolve any missed script.
2. Transcripts were coded using NVivo software (Version 11 QSR International Pty Ltd. NVivo qualitative data analysis software, 2015) to identify important components in the data which were associated with physical activity behavior and social networks.
3. Coding was conducted independently by two researchers (SM and RH) to establish inter-rate reliability, who produced two lists of codes and multiple discussions were held to discuss coding frameworks.
4. The framework was reviewed until both researchers were satisfied with the themes.
5. Three major themes pertaining to the key findings identified in phase 1, and two additional themes were identified from the data.
6. Codes were concentrated into sub-themes and analyzed, with illustrative quotes relating to each sub-theme.

## Results

### Phase 1: quantitative phase

#### Participant characteristics

A total of 529 participants from 23 schools (48.6% female) in Northern Ireland (mean age 14.38 years, SD 0.32) were included in phase 1 (Table 2). The mean physical activity behavior of all participants (PAQ-C summary) was 2.78, SD 0.67 (min 1, max 5) and males had a higher mean physical activity behavior (3.01, SD 0.67) compared to females (2.54, SD 0.58). Previous research reflected similar findings for adolescent males’ and females’ PAQ-C summaries, and suggested PAQ scores greater than 2.9 for males and greater than 2.7 for females, were necessary for protecting against adverse metabolic health [60]. This suggests the physical activity behavior of the participants in this study fell short of the recommendations, and males had greater physical activity behavior compared to females, in expected. Males also had a greater mean out-degree (5.96, SD 3.47), or number of nominations given (min 0, max 10), compared to females (5.63, SD 3.30).

**Table 2:** Participant characteristics (Phase 1)

	Males (*n* = 272)				Females (*n* = 257)				Overall (*n* = 529)			
Participant characteristics	Mean	S.D.	N		Mean	S.D.	N		Mean	S.D.	N	
			Valid	Missing			Valid	Missing			Valid	Missing
Age (years)	14.39	0.30	272	0	14.38	0.34	257	0	14.38	0.32	529	0
Female (%)									46.80			
Physical Activity												
Individual PAQ-C summary mean	3.01	0.67	268	4	2.54	0.58	257	0	2.78	0.67	525	4
Friend group PAQ-C summary mean	3.05	0.40	233	39	2.57	0.33	223	34	2.81	0.44	456	73
Best friend’s PAQ-C summary	3.05	0.66	221	51	2.51	0.58	206	51	2.79	0.68	427	102
Out-degree	5.96	3.47	272	0	5.28	3.08	256	1	5.63	3.30	528	1
In-degree	6.00	3.17	272	0	5.22	2.63	257	0	5.62	2.95	529	0

Individual PAQ-C summary mean is derived from nine items, each scored on a 5-point scale to assess general physical activity levels

Friend group PAQ-C summary mean is derived from the mean PAQ-C summary means of participant’s nominated friends

Best friend PAQ-C summary mean is derived from the participant’s first nominated friend’s PAQ-C summary mean

#### Correlation between individual and friend physical activity behavior

Pearson’s correlation coefficient assessed the relationship between individual physical activity behavior and the mean physical activity behavior of their friend group and best friend. The results (Table 3) showed that there was a statistically significant linear relationship between individual- and friend group-level physical activity behavior, but the strength of the correlation was moderate for males (0.43, *p* < 0.0001) and weak for females (0.26, *p* < 0.0001). Similarly, the strength of the correlation between individual- and best friend- physical activity behavior was moderate for males (0.40, p < 0.0001) and weak for females (0.28, *p* < 0.0001). All correlations were positive, indicating that pupils with higher levels of physical activity nominate best friends with higher levels of physical activity and groups of friends with higher physical activity.

**Table 3:** Pearson’ correlation coefficient showing the strength of the relationship between individual physical activity and their friend group and best friend’s physical activity (phase 1)

	Individual PAQ-C summary mean			
	Female (n)	Sig. (p)	Male (n)	Sig. (p)
Friend group PAQ-C summary mean	0.26 (223)	< 0.0001	0.43 (233)	< 0.0001
Best friend’s PAQ-C summary mean	0.28 (206)	< 0.0001	0.40 (221)	< 0.0001

#### Association between individual physical activity and friendship network characteristics using multiple regression

Table 4 presents the results of the multiple linear regression analysis that investigated the association between friendship social networks and physical activity behavior, by sex.

**Table 4:** Multiple regression analyses of peer social network factors with individual physical activity level (phase 1)

Male Individual PAQ-C summary mean (*n* = 221)							Female Individual PAQ-C summary mean (*n* = 205)					
	Model 1 (95% CI)	P	Model 2 (95% CI)	P	Model 3 (95% CI)	P	Model 1 (95% CI)	P	Model 2 (95% CI)	P	Model 3 (95% CI)	P
Friend group PAQ-C summary mean	***0.72 (0.53, 0.92)***	**< 0.001**	***0.46 (0.20, 0.72)***	**< 0.001**	***0.46 (0.14, 0.78)***	**0.007**	***0.45 (0.23, 0.67)***	**< 0.001**	0.24 (−0.04, 0.53)	0.09	0.25 (−0.09, 0.58)	0.14
Best friend’s PAQ-C summary mean	***0.41 (0.29, 0.54)***	**< 0.001**	***0.21 (0.05, 0.37)***	**0.01**	0.21 (−0.04, 0.45)	0.09	***0.28 (0.15, 0.41)***	**< 0.001**	***0.21 (0.05, 0.37)***	**0.01**	***0.21 (0.02, 0.40)***	**0.03**
Out-degree	***0.04 (0.02, 0.06)***	**0.001**	0.02 (−0.02, 0.05)	0.38	0.02 (−0.03, 0.06)	0.47	***0.02 (−0.01, 0.04)***	**0.02**	0.00 (−0.03, 0.03)	0.91	0.00 (0.06, 0.06)	0.95
In-degree	***0.05 (0.02, 0.07)***	**< 0.001**	0.01 (−0.02, 0.04)	0.44	0.01 (−0.02, 0.04)	0.42	0.00 (− 0.03, 0.03)	0.91				

Model 1: independent variables (friend group PAQ-C summary mean, best friend’s PAQ-C summary mean, out-degree and in-degree) were investigated as potential predictors of individual PAQ-C summary mean (dependent variable)

Model 2: all four variables added to the model simultaneously

Model 3: adjusted for clustering using STATA version 14.0

#### Association between physical activity behavior and friendship social networks in males

Model 1 (univariate analyses) showed all variables to be statistically significant (at *p* = 0.05), with friend group physical activity the strongest predictor of individual physical activity behavior (0.72, *p* < 0.001), followed by best friend’s physical activity behavior (0.41, p < 0.001). Out-degree (0.04, *p* = 0.001) and in-degree (0.05, p < 0.001) were statistically significant in this model, although were associated with very small incremental increases in individual physical activity behavior.

Model 2 (multivariate analyses) included all predictor variables that were statistically significant from univariate analyses. Only friend group physical activity behavior (0.46, *P* < 0.001) and best friend’s physical activity behavior (0.21, *P* = 0.01) remained statistically significant predictors of individual physical activity behavior.

Model 3 adjusted for classroom level social network clustering. Friend group physical activity behavior remained a statistically significant predictor of male adolescent physical activity behavior (0.46, *p* = 0.007).

#### Association between physical activity behavior and friendship social networks in females

Model 1 (univariate analyses) showed friend group’s physical activity behavior was most strongly associated with individual physical activity behavior (0.45, *p* < 0.001), followed by best friend’s physical activity behavior (0.28, *p* < 0.001) and out-degree (0.02, *p* = 0.02). In-degree was not statistically significant (*p* = 0.91), and therefore was not entered into Model 2.

Model 2 (multivariate analyses) showed only best friend’s physical activity behavior was a significant predictor of individual physical activity behavior (0.21, *p* = 0.01), which remained statistically significant (0.21, *p* = 0.03) after adjusting for classroom level social network clustering (Model 3).

The findings of this phase identified a number of key findings that were further investigated using qualitative exploratory methods:

1. There was a weak to moderate correlation between individual physical activity behavior and the physical activity behavior of friends, with a stronger correlation for males than females. This indicates that *friends have similar physical activity behaviors*;
2. *Friendship social networks may impact differently on early adolescent male and female physical activity behavior.* The physical activity behavior of the friend group was significantly associated with male physical activity behavior, but not female physical activity behavior, and the physical activity behavior level of the best friend was significantly associated with female, but not male physical activity behavior;
3. *Popularity and sociability were not associated with physical activity behavior*, as in-degree and out-degree were not shown to be significantly associated with physical activity behavior.

### Phase 2: qualitative phase

#### School characteristics

The sample of schools included in the qualitative phase ranged in terms of their demographic characteristics and were reflective of the larger sample of schools in phase 1 (i.e. including single-sex and mixed-sex education, deprivation levels, location and school type (grammar or secondary) (Table 1)).

The three key findings that were identified in phase 1 were explored further during focus group discussions:

1. *Friends have similar physical activity behaviors;*
2. *Friendship social networks may impact differently on early adolescent male and female physical activity behavior;*
3. *Popularity and sociability were not associated with physical activity behavior*.

The thematic analysis of focus group data added understanding about a broader social network and set of environmental influences (i.e. non-classroom peers and family) as represented by the following themes:

4. *Social norms; and*
5. *External factors (which impact upon the relationship between adolescent physical activity behavior and social networks)*.

Conceptually, the findings from the quantitative phase 1 and their subsequent exploration and analysis during the qualitative phase of the study combined with the findings from the bottom-up thematic analysis of focus group data are presented as five main ‘themes’ (and sub-themes).

#### Theme one: friends have similar physical activity behaviors (identified in phase 1)

Quantitative analysis from phase 1 identified a significant correlation between individual and friend physical activity behavior. Although the correlation was weak for females and moderate for males, it suggested a similarity between friends’ physical activity behaviors for both sexes. Thematic analysis of focus group discussions further explored the role of friends for determining individual physical activity behavior, and identified an important emphasis on adolescents’ own immediate, direct friendship social networks (i.e. their friends they spent time with inside school, outside of school and through sports and clubs, or were in the presence of on a daily basis) and the resulting impact they had on their physical activity behavior. Four sub-themes emerged: (A) friendship formation through physical activity behavior; (B) spending time with friends; (C) negative influence from friends; and D) utilizing peer networks to encourage physical activity behavior.

#### Sub-theme A: friendship formation through physical activity behavior

Participants did not consciously aim to select friends who reflected similar physical activity behaviors. Rather, personality was the driving factor behind friendship formation. Females, in particular, did not regard having similar physical activity behaviors to their friends as an important quality for friendship formation, *‘just who they are, as a person’ – female – Focus Group (FG)2*. Having different likes or dislikes in regards to physical activity behavior did not have negative implications on friendship formation, *‘it’s not like you ask them do they like hockey, and if they don’t you don’t say ‘oh, I’m not going to be your friend’ – female - FG2.* However, participants acknowledged that common physical activity behaviors (i.e. sporting or spare time interests) were a good foundation for friendship formation and such friendships were more stable, *‘if you do have a sport in common then obviously it makes you more friendly, you have same interests and you become better friends, but it’s not like at first if you don’t do whatever, I’m not going to be your friend’ – female - FG2.* Furthermore, participation in sports’ teams or clubs provided friendship opportunities, *‘all of my friends would probably be on the rugby team’ – male (rugby player) FG1.*

#### Sub-theme B: spending time with friends

Many participants did not purposively intend on being active, but spending time with their friends often resulted in informal physical activity. Engaging in physical activity behavior was a by-product of spending time with friends, which differed for males, *‘I just go around to their house for a ‘kick about’ (slang term for football)’ – male - FG5;* and females, *‘me and (female named) go out sometimes and walk around (local area named) and walk down to the park and stuff, just talking’ – female - FG2.* Participants described physical activity behavior with friends as more fun, enjoyable and less of a chore compared to being alone or with peers they did not know as well, *‘I’d rather run about with my mates than with people who are really sporty, because they encourage me as well’ – male - FG4.* Shared physical activity behavior presented opportunities for friends to spend time together, *‘my friend started going to the gym once a week, so I go with her now; at the weekends we can go together’ – female - FG2.* In school, participants enjoyed Physical Education classes more when they were able to be with their friends, *‘you have to get into groups and partners quite a lot, and if you’re with your friends it’s a lot more fun to do’ – female - FG2.*

#### Sub-theme C: negative influence from friends

Friends had the potential to discourage physical activity behavior for both males and females, ‘s*ay you wanted to go out and play football and maybe two or three don’t want to play it and then everyone ends up not doing it or it’s too cold or something’ – male - FG1.* Whilst participants enjoyed physical activity with friends, they were frustrated when it interrupted their free time to spend with friends. Missing out on spending time with friends due to sport commitments was off-putting and a negative side effect to physical activity which discouraged them from continuing, *‘it would make you not want to go as much and then you might start missing it every so often and then... You just stop going’ – female - FG3.* Friends also had the ability to instigate negative, risky behaviors, *‘ … Like if you see one of your best players going out on the “sesh” (local slang term for drinking alcohol), all the other players would be like “oh he can do it, so I can do it”’ – male - FG5.*

#### Sub-theme D: utilizing peer networks to encourage physical activity behavior

Participants explored ideas for encouraging their friends and each other to be physically active. They suggested exploring what they might like to do and encouraging them to try new activities, *‘you would have to try and find out their suggestions for more clubs or something like that or what they enjoy’ – male - FG1.* Participants discussed adopting supportive techniques and strategies to try to engage their friends in physical activity behavior, through enticing them with opportunities to spend time with friends, *‘tell them the other friends are coming as well and that it will be good’ – male - FG1.* Participants discussed competitiveness with friends in physical activity and sport. Between friends, light-hearted competitiveness was motivational and helped to encourage physical activity behavior, for both males, *‘there is always competitiveness between all of us, but we’d always work together as well’ – male - FG1* and females, *‘you have competition between your friends, you want to be better than them but you support them so that they can get better, or just as good as you’ – female - FG5.*

#### Theme two: friendship social networks may impact differently on early adolescent male and female physical activity behavior (identified in phase 1)

Findings from phase 1 showed a significant association between males’ physical activity behavior and the physical activity behavior of their friend group but not their best friend; whereas females’ physical activity behavior was significantly associated with the physical activity behavior of their best friend, but not their friend group within the classroom setting. Thematic analysis allowed for exploration into potential differences in the role of social networks for physical activity behavior in males and females. Three sub-themes emerged: A) direct social support networks; B) school Physical Education class structure and gender inequality and (C) influence on physical activity behavior from the opposite sex.

#### Sub-theme A: direct social support networks

Direct social network members (i.e. friends, or individuals who are more intimately tied within a social network) provided social support for physical activity behavior. Females discussed how friends could positively influence physical activity behavior, through encouraging engagement, *‘if you didn’t really want to go to hockey and your friend was like “please go” you’d probably end up going … they would encourage you to go to more practices and to keep it up and stuff’ – female - FG2.* Friendship social networks can provide a stable, comfortable environment in which adolescents feel more comfortable participating in physical activity. This was particularly evident for females, who were reluctant to participate without the support of friends, *‘you feel like if you’re going on your own then you’re worrying about it all the time, but if your friends are going it’s like “ah, that’s grand; they’re doing that so I’ll just do it”’ – female – FG3.* For males, friends were motivational, *‘being with your mates motivates you more. It actually makes you better’ – male - FG4*. Males also found the competitive nature of friends could provide motivation and encouragement, *‘if there is someone better than you then you can go and … try your hardest to be better than them’ – male - FG4.*

#### Sub-theme B: school physical education structure and gender inequality

Participants discussed participation and engagement in Physical Education during school. Unlike other schools within the United Kingdom, most Northern Irish schools are governed by religious background (Protestantism and Catholicism) and school education type (secondary or grammar selective system). These factors determine the Physical Education or sports that will be offered at the school that are traditionally aligned, which can result in the formation of segregated sports networks and a limitation on physical activity choice. The restrictions limiting free choice in sport was discussed mainly by male participants, *‘all the rugby coaches say that... Football’s a terrible sport...that you knew it was a rugby school when you came here...’ – male - FG1.* In the one mixed sex group, females were vocal in highlighting issues of sexism, as preference was given to the males when it came to shortage in Physical Education space or attention from teachers, *‘I find our school to be a bit sexist with PE, because the males get to do whatever. Like last year, the boys were outside playing football while the girls were stuck in and made to make up a random dance. That’s not fair on the girls if they want to go out and play football or something’ – female - FG5.* Females were very conscious of the assumption from teachers that males were more interested and capable of sport or physical activity, and described this negatively, *‘They might think the boys are a lot more into sports ...girls are less interested than the boys’ – female - FG5.* Females also perceived males to be more important in the eyes of coaches*, ‘they’re [males] more important’ – female – FG5.*

#### Sub-theme C: influence on physical activity behavior from the opposite sex

This study included one mixed sex group, males (*n* = 4) and females (*n* = 5). Discussion of mixed-sex influence on physical activity behavior identified some key points for engagement and participation. For females, physical activity behavior in a mixed sex group was motivational and encouraging as they had to push themselves to keep up with the males, *‘I think it pushes you a bit harder’ – female - FG5.* However, males did not share the same views and were conscious that mixing the sexes could lead to distraction, *‘you see some boys who could be absolute “rods” (local slang term to indicate ‘showing off’) when they come near females, all they will do is sit and chat and they won’t get on and play, so that’s why I think they don’t mix them, because they would literally just sit and chat for the whole lesson’ – male - FG5.*

#### Theme three: popularity and sociability were not associated with physical activity behavior (identified in phase 1)

Findings from phase 1 suggested that classroom popularity and sociability were not significant predictors of adolescent physical activity behavior. Thematic analysis further explored the impact of popularity and sociability on physical activity behavior.

Participants discussed the association between popularity and physical activity behavior. Neither males nor females were supportive of more active peers being more popular, but instead suggested that they were more well-known due to their sporting achievements, *‘they’re not really popular, are they? It’s not like they’ve become really popular because they’re on that team. But people would know them’ – female - FG2.* The concept of being well-known was frequently associated with being part of a sports team or club at school, and being recognized and admired by others, *‘you’ve won lots of awards so people would know you from assembly. You would be at the front in assembly and your name would be read out with this big trophy, so people would know you from receiving that award or whatever’ – female - FG2.* The concept of being well-known through sports in school was attractive to participants, as this provided the benefits of winning and being noticed by older peers, *‘if you win an award or if you’re on a team older than you, then the older team would know you and talk about you because you’re so good and you’ve been brought up a year to play for them or something’ – female - FG2.* Participants suggested highly active peers had more friends, due to making friends through sport, *‘they probably have more friends through sport’ – female - FG2.* However, they were dismissive of associating highly active peers with being more popular, *‘you’re your own person, I wouldn’t look up to someone who’s just good at football or good at rugby...’ – male - FG1.*

#### Theme four: social norms

Thematic analysis of focus group discussions allowed for exploration of broader social network influences impacting on physical activity behavior that could not be identified through quantitative analysis. Social norms was identified as a theme, as participants discussed their perceptions of peers who were outside of their direct friendship social networks. These perceptions were of collective influence from the broader social environment, formed from adolescents’ indirect network ties to individuals they did not actively seek to spend time with. Whilst such individuals were not considered to be members of the participants’ own friendship social networks, they were part of the broader social environment (i.e. school year peers). Although participants did not spend as much time in the presence of these peers as they did with their own friends, or did not have a personal relationship with them, they were influenced by them indirectly, through perceptions of their thoughts and actions. Two sub-themes were identified: (A) perceptions of highly active and highly inactive peers; and (B) utilizing indirect peer social networks to encourage physical activity behavior.

#### Sub-theme A: perceptions of highly active and highly inactive peers

Participants discussed the personalities and qualities of peers they perceived to be highly active and highly inactive. Generally, active peers were perceived to be positive and healthy individuals, *‘energetic people’* – male - FG1. Females were respectful of the time such individuals put into their sport or activity, *‘they’re always really dedicated’* – female - FG3. However, many participants had a negative perception about highly active peers. Females perceived such individuals to regard themselves to be better than those who were less active, *‘I think people who take part in sports nearly hold themselves higher to everyone else anyway’ – female - FG3.* This left them feeling embarrassed and intimidated in comparison, *‘sometimes they can be wild (local slang term for extremely) intimidating, because if someone is really good at something and you’re not, you feel like you have to be like on their level. Have to impress them’ – female - FG3.* Males held similar views of frustration, however they showed signs of envy and anger towards highly active peers, describing them as *‘show-offs!’ – male - FG4.* Males’ perceptions of inactive peers were derogatory, as they focused on the association between lack of social interaction and poor social skills with being inactive, *‘they’re socially inept’ – male - FG1*. This was also associated with staying indoors to play sedentary computer games, *‘they [inactive adolescents] are all just playing their DS’ – male - FG1.* Females did not share the males’ derogatory viewpoints of inactive peers and were more empathetic and understanding to why some individuals did not want to participate in sporting activities such as PE, *‘they feel “oh, I can’t go over to that group because they know what they’re doing and I don’t”. They’re embarrassed’ – female - FG3.*

#### Sub-theme B: utilizing indirect peer networks to encourage physical activity behavior

Participants discussed strategies to engage peers from their wider social network (i.e. school year-group peers) in physical activity behavior. Some participants suggested motivating others by challenging them, *‘you wouldn’t say something negative straight to their face, you would try and inspire them, tell them what they need to do to build up towards it’ – male - FG1.* Participants were conscious that being too serious would be off-putting for peers and having fun or not being overly competitive would make peers feel more at ease to participate, *‘sometimes people can be embarrassed if they haven’t played the sport before and they’re in a team with people who have been doing it for years. You have to make it like it’s only PE, it’s not really - Competitive’ – female - FG3.* Other participants suggested an empathic approach to engaging others through inspiring them, *‘you try and bring them on’ – female - FG5 and encouraging them, ‘you say “you’ll get the next ball, you’ll get the next one, you know...” you’re always keeping them going’ – male - FG5.*

Participants discussed the effectiveness of mixing together physical activity levels to encourage those who are less active. Participants suggested that mixed physical activity level teams in Physical Education are opportunistic for and help less active peers to improve, *‘the way it’s mixed, say there are some of us not as good as another person, the person who is not as good might get motivated to try and be better’ – male - FG4.* However, not all participants were supportive of mixed ability sessions as they were concerned that less active peers would put little effort in to the activity, *‘you’re not going to have to full class where everyone is going to want to participate in PE (Physical Education) and then everyone is getting assessed and they won’t put the effort in and it just lowers everybody then’ – female - FG3.*

#### Theme five: external factors (which impact upon the relationship between adolescent physical activity behavior and social networks)

Thematic analysis identified other external factors which could not be investigated through quantitative methods. These factors impacted the relationship between adolescent physical activity behavior and social networks, but could not be controlled or manipulated (such as seasonal dependency of outdoor physical activity). The findings suggested that external factors impact indirectly on individual physical activity behavior engagement, enjoyment, participation and social networks. Three sub-themes were identified: (A) the influence of teachers and coaches; (B) sports team selection and (C) weather conditions.

#### Sub-theme A: the influence of teachers and coaches

Participants emphasized that highly active individuals were given more attention from teachers or coaches. This was especially off-putting for individuals who enjoyed a sport but were not on the team, as they felt their presence was less important in comparison, *‘it’s more like if you’re not on the team... you can still go but you don’t get much attention paid to you. It’s normally just the people that are on the team... No one else’ – female - FG2.* Participants identified favoritism from teachers for ‘sporty’ individuals over the rest of the pupils, *‘they get priority’ – female - FG3.* Some participants referred to their coaches or teachers’ motivational strategies as poor, due to a strict and harsh approach, *‘they’re encouraging them, but in a harsh way … – male - FG5.* They identified differences between peer and coaches’ motivational strategies, *‘the coaches are there to shout; you’re there to encourage, as such’ – male - FG5.*

#### Sub-theme B: sports team selection

The exclusivity and selection procedure of sports teams was off-putting for many participants, even if they enjoyed the activity. These close-knit, exclusive networks made participants feel less welcome to attend the training or club if they were not be part of the team, ‘*you’d feel a bit out of it if you went, I think’ – female - FG2* and *‘if you don’t get onto a team people would just quit’ – female – FG2.*

#### Sub-theme C: weather conditions

Despite acknowledging that when the weather was good it enabled individuals to go outside and be physically active, weather was mainly described as an inhibitor to physical activity behavior, due to seasonal dependencies of many sports or activities, *‘if you’re in a club then it has to be on’ – male - FG1.* Seasonal dependencies of activities were associated with restricting time spent with friends or shared activities with friends, *‘during summer I would be with my school friends, during winter I’d be with my football ones, because there is no football ones that go here [school]. In summer I’d be able to go into [local area named] or somewhere because it’s nice weather and you can make it up there, but in winter I have to stay about [local area named] because usually the roads are too dodgy to go to [local area named] – male – FG5.*

## Discussion

**Fig. 1:**
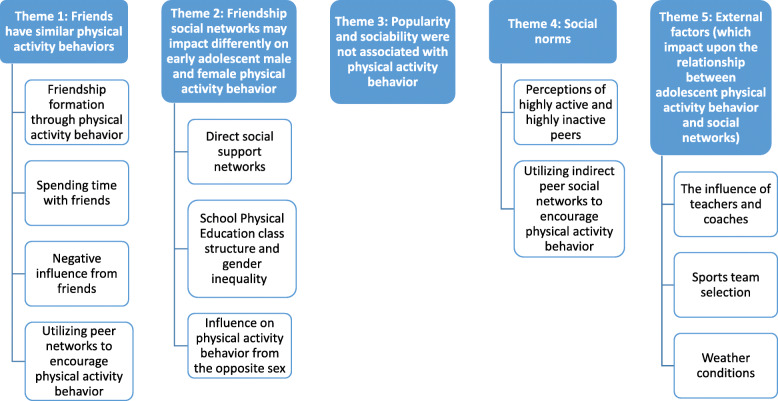
Themes and associated sub-themes emerging from the role of social networks for adolescent physical activity behavior (phases 1 and 2)

This study investigated the relationship between the social networks of young people (aged 13–15 years) and their physical activity behavior. Findings from phase 1 (quantitative) highlighted important differences between males and females regarding adolescent friendship social networks and physical activity behavior. Males’ physical activity behavior was associated with the physical activity behavior of their friend group and females’ physical activity behavior was associated with their best friend’s physical activity behavior. Phase 2 (qualitative) allowed for greater in-depth investigation of the findings, alongside the investigation of the broader role of social networks (Fig. 1). The investigation of the interplay between the findings from each phase of the inquiry provides a more comprehensive view of social network influences. It is known that adolescents typically spend much of their time in the company of peers [26]. However, it is important to consider time spent with peers who lie beyond the bounded classroom network setting (i.e. time spent with peers in neighborhood settings or leisure activities). Therefore, investigation of the interplay between the findings from each phase allowed for a more well-rounded synthesis of the social network influences impacting on physical activity behaviors. Furthermore, it should be acknowledged that the themes and sub-themes do not exist in isolation, as social networks impact physical activity behavior through multiple levels within a complex system including intertwining biological, behavioral, social and economic factors [61]. There is a need to recognize that there are interconnections between the themes and sub-themes, however, it is beyond the scope of this study to begin to explore these interconnections more in-depth. Furthermore, social networks have the potential to encourage or discourage physical activity behavior in adolescents. Incorporation of social network concepts within physical activity behavior change intervention design may encourage more promising intervention effects [21, 30]. The findings highlight that peer social networks may impact physical activity differently for adolescent males and females. Acknowledgement of these differences in the association between social networks and physical activity for either sex may allow for social network interventions to be tailored, leading to more effective behavior change implementation.

### Implications for physical activity behavior change interventions for males

Integration of findings from both phases suggests that physical activity behavior change interventions in males may benefit from a social network component based on a segmentation approach [62], which targets physical activity behavior change at specific groups of individuals. For example, physical activity behavior change may be more likely to occur due to changing social norms, reinforcement through group behavior and co-operation between group members to achieve goals [63]. The findings from Phase 1 suggested that boys’ physical activity is similar to the activity of their friend group, and the qualitative exploration supported the idea that adolescent males are more encouraged to engage in physical activity behavior as a by-product of spending time with their friends and having fun with friends, without the stress of high-level sport commitment. This accords with previous qualitative studies in which the presence of friends and lack of competition were frequently mentioned as motivational factors [64].

### Implications for physical activity behavior change interventions for females

Whilst a segmentation approach may be effective in males, the findings are more supportive of a dyad-based approach (dyads represent a pair of nodes in the network [65]) for encouraging adolescent females’ physical activity behavior, as females’ physical activity behavior was predicted by their best friend’s physical activity behavior only in Phase 1. Furthermore, in Phase 2, females expressed the importance of social support and encouragement from friends. Females were more likely to engage in physical activity behavior when they were in the presence of a close friend. Previous research supports the importance of social support and positive peer relationships for encouraging physical activity behavior [66–68]. In particular, a previous study found adolescent females who frequently took part in physical activity with their best friend obtained higher levels of physical activity compared to females who did so less frequently [69]. Findings from this study point to the need for interventions to focus on building support for physical activity behavior and encouraging pairs of friends to be active together [69]. Interventions that give adolescent females the opportunity to be active with a chosen other, such as a best friend, may encourage participation through increased social support, as has been recommended in previous literature [70]. It is particularly important to target female physical activity at this age, given the particularly steep reduction in physical activity levels of adolescent females compared to males [10]. Findings from phase 2 highlighted possible pathways through which direct support from friends positively influenced physical activity behavior, through the provision of motivation, encouragement and stability, allowing adolescents to feel comfortable participating in physical activity. This was particularly important for females, as they felt less awkward and embarrassed being active with their friends, whereas males associated physical activity with friends with having fun.

### Implications for future research

Previous research has highlighted that influences on physical activity at the school and family level, and extracurricular sport participation, are weaker in adolescent females compared to males [71]. Furthermore, research has suggested that understanding the social environments in which an intervention is delivered can allow it to be tailored, thereby potentially increasing long-term effectiveness [72]. For the development of effective complex physical activity interventions, longitudinal studies are required, to explore the relationship between social networks and physical activity behavior over time, identifying how physical activity behavior spreads throughout a social network or influences friendship patterns. Greater understanding of specific network processes which impact physical activity behavior over time would be useful for the development of complex interventions which are tailored to the population.

Therefore, questions remain regarding the generalizability and transferability of the results to other societies worldwide. Ultimately social mechanisms of different types can be perceived to be the ‘causal forces’ that generate desirable intervention outcomes. These social processes, only partially observable themselves, are dependent on relationships and interactions, and therefore need to be investigated further with regards to behavior change, especially as they may be highly dependent on the social context [73].

### Strengths and limitations

The sampling strategy adopted for phase 1 utilized an 80% network completeness eligibility criterion to ensure robustness of the data and inference. This figure was taken from common practice for Stochastic Actor Based Models [51]. However, there is currently no guidance for cross-sectional social network data. To the best of our knowledge, there is no validated method of measuring social networks and no other guidance for standardized criteria for complete network data, therefore a strength of this study is the high quality network data utilized.

A limitation to this study may arise from our calculation of the mean physical activity of the friend group. In this calculation, we included the best friend, as they are an important individual within the friend group. This differs from previous literature, in which the authors chose to remove the best friend from the friend group analyses [74] as it may infer issues surrounding high correlation between variables. Thus, the data were tested for multicollinearity and no issues were identified (Variance Inflation Factor scores were below 1).

This study was limited to a single UK region, however we included a broad range of sociodemographic characteristics. It was only possible to measure classroom friendship networks during phase 1 and therefore findings may not be generalizable outside of the classroom setting. However, it should be acknowledged that adolescents spend a considerable amount of time in school and timetabled sport occurs within these settings. Furthermore, qualitative investigation provided insight into longer-term trends of networks and their impact on behavior [75] and allowed for the exploration of social network concepts outside of the bounded network.

To date, the adoption of a multi methods approach within the field of social networks has been slow [76] but qualitative approaches can augment our understanding of the mechanisms by which social networks impact behavior [77]. Due to the dependent nature of structures and social processes, the fusion of quantitative and qualitative methods allows for a richer and more nuanced perspective on such mechanisms [78]. However, this is not a panacea, as multiple mechanisms may operate simultaneously and the complex causal relationships between social networks and behavioral outcomes are only ever glimpsed partially [77]. It should be acknowledged, however, that the specific qualitative approach utilized in this study may have introduced some level of bias, as the topic guide was developed to further explore quantitative findings. Nevertheless, the topic guide included broader questions, to explore the nuances of the relationship between social networks and physical activity outside of the quantitative findings and allow for voicing of other issues.

This study included a greater proportion of least deprived schools compared most deprived schools (87 and 80% of schools were ‘least deprived’ in phases 1 and 2, respectively). This may raise issues regarding generalizability of the results, however the overall sample included in both phases included a range of grammar and secondary education schools, from locations throughout Northern Ireland, and included an almost equal split of males and females (46.8 and 51% female in phases 1 and 2, respectively). However, focus group discussions were mainly with single-sex groups (*n* = 4) and only one group was mixed-sex. Although the overall inclusion of focus group participants was almost equally split, the study results are limited by the inclusion of mainly single sex schools and therefore future research would benefit from additional exploration of mixed sex schools to further explore sex differences.

## Conclusions

Findings from this multi-method analysis provided support for tailored social network approaches to physical activity behavior change interventions for adolescents. Integration of phases 1 and 2 showed a significant association between individual and friend group’s physical activity behavior for males, and individual and best friend’s physical activity for females. This provided some support for group-based (segmentation) approaches for physical activity behavior change for males and dyad-based approaches for females. Phase 2 allowed for broader exploration of social networks and physical activity behavior. Peer social networks may encourage or discourage adolescent physical activity behavior through the interaction of multiple direct and indirect social network influences. Thematic analysis of phase 2 focus group discussions highlight a complex system of intertwining social network influences, which have the potential to encourage or discourage individuals’ physical activity behavior, and could impact the effectiveness of physical activity behavior change interventions. Further research is needed to explore how such concepts could best be utilized within physical activity behavior change interventions.

## Supplementary Information

**Additional file 1.**

## Data Availability

The datasets used and/or analyzed during the current study are available from the corresponding author on reasonable request with permission from the School of Social Sciences, Education and Social Work, Queen’s University Belfast, Belfast, Northern Ireland, UK and the Centre for Public Health, School of Medicine, Dentistry and Biomedical Sciences, Queen’s University Belfast, Belfast, Northern Ireland, UK.

## Abbreviations

EAT-2010: Eating Among Teens-2010
MVPA: Moderate to vigorous physical activity
PAQ-C: Physical Activity Questionnaire for Children
UK: United Kingdom
WiSe: Wellbeing in Schools
