# Supplementary material for: A multi-method exploration into the social networks of young teenagers and their physical activity behavior
Source: BMC Public Health. 2021 Jan 7;21:77. doi: 10.1186/s12889-020-10081-0 (PMC7792163; doi:10.1186/s12889-020-10081-0)
Supplement: Supplementary file 1 — Additional file 1. [file 12889_2020_10081_MOESM1_ESM.docx]

# Appendix File 1

Table 1: Demographic characteristics of schools included in WiSe wave 2 and the Phase 1 sample

|  | WiSe wave 2 | | Phase 1 sample | |
| --- | --- | --- | --- | --- |
|  | **N** | **%** | **N** | **%** |
| **School Type** |  |  |  |  |
| Grammar | 60 | 68.2 | 17 | 73.7 |
| Secondary | 28 | 31.8 | 6 | 26 |
| **NI Education Board (location)** |  |  |  |  |
| Belfast | 9 | 10.2 | 3 | 13 |
| North-East | 12 | 13.6 | 3 | 13 |
| South-East | 15 | 17 | 3 | 13 |
| Southern | 26 | 29.5 | 6 | 26 |
| Western | 26 | 29.5 | 8 | 35 |
| **County** |  |  |  |  |
| Antrim | 20 | 22.7 | 6 | 26.1 |
| Armagh | 5 | 5.7 | 1 | 4.3 |
| Down | 25 | 28.4 | 5 | 21.7 |
| Fermanagh | 6 | 6.8 | 2 | 8.7 |
| Londonderry | 16 | 18.2 | 5 | 21.7 |
| Tyrone | 16 | 18.2 | 4 | 17.5 |
| **Management** |  |  |  |  |
| Voluntary | 25 | 28.4 | 13 | 56.6 |
| Controlled | 24 | 27.3 | 5 | 21.7 |
| Roman-Catholic Maintained | 31 | 35.2 | 5 | 21.7 |
| Grant-Maintained Integrated (GMI) | 8 | 9.1 | 0 | 0 |
| **Sex** |  |  |  |  |
| Mixed | 65 | 73.9 | 15 | 65.3 |
| All-boys | 11 | 12.5 | 5 | 21.7 |
| All-girls | 12 | 13.6 | 3 | 13 |
| **Religion** |  |  |  |  |
| Mainly Protestant | 35 | 39.8 | 10 | 43.5 |
| Mainly Catholic | 45 | 51.1 | 12 | 52.2 |
| Integrated | 8 | 9.1 | 1 | 4.3 |
| **Deprivation** |  |  |  |  |
| Most Deprived (>37.5% eligible for FSM) | 30 | 34.1 | 3 | 13 |
| Least Deprived (<37.5% eligible for FSM) | 58 | 65.9 | 20 | 87 |

*Northern Irish schools differ from schools in the UK or Republic of Ireland. A selection exam during the final year of primary school determines a Grammar or Secondary post-primary school place. There are five board locations of schools (see above) which covers six counties (see above). Schools are governed by four main types of management: Voluntary, Controlled, Roman-Catholic Maintained and GMI. Children are eligible for free school meals (FSM) if their parents receive income support, or their net earnings do not exceed £14,000/year.*

*(Dep. of Education, 2018a; Dep. of Education, 2018b)*
